# Copeptin and albumin in children with undernutrition

**DOI:** 10.3389/fped.2026.1902840

**Published:** 2026-10-07

**Authors:** Halil Uğur Hatipoğlu, Ahsen Güler, Murat Elevli

**Affiliations:** Department of Pediatrics, University of Health Sciences, Hamidiye Faculty of Medicine, Istanbul Haseki Training and Research Hospital, Istanbul, Türkiye

**Keywords:** albumin, biomarker, case-control study, copeptin, pediatric undernutrition, ROC analysis

## Abstract

**Background:**

Anthropometric assessment remains the cornerstone of the identification and classification of pediatric undernutrition, whereas the role of laboratory biomarkers is less well established.

**Objective:**

To evaluate serum copeptin in children with undernutrition and to compare its discriminatory performance with that of serum albumin.

**Methods:**

This single-center cross-sectional case-control study included children aged 1 month to 18 years with undernutrition and healthy controls recruited from the same clinical setting. Serum copeptin concentrations were measured by enzyme-linked immunosorbent assay. Receiver operating characteristic analysis and multivariable logistic regression were performed. Age-stratified and sensitivity analyses were also conducted.

**Results:**

A total of 98 children were included: 44 with undernutrition and 54 healthy controls. Serum copeptin concentrations were significantly higher and serum albumin concentrations were significantly lower in children with undernutrition than in healthy controls (both *p* < 0.001). In ROC analysis, copeptin yielded an area under the curve of 0.729 (95% CI 0.622–0.835), whereas albumin yielded an area under the curve of 0.726 (95% CI 0.618–0.833), with no significant difference between the two ROC curves (DeLong *p* = 0.977). In multivariable analysis adjusted for age and sex, both copeptin and albumin remained independently associated with undernutrition. A combined copeptin–albumin model showed higher discrimination within the study sample.

**Conclusion:**

Serum copeptin is elevated in children with undernutrition. Although it does not outperform albumin as a standalone biomarker, it may provide complementary information when interpreted alongside albumin.

## Introduction

Child undernutrition remains a major global health challenge. The 2025 joint UNICEF/World Health Organization (WHO)/World Bank estimates indicate that wasting continues to affect millions of children worldwide, underscoring the need for timely recognition and clinically practical risk stratification in pediatric care (1).

In pediatric practice, anthropometric assessment remains the cornerstone of the identification and classification of undernutrition. Current pediatric malnutrition frameworks emphasize standardized age-appropriate indicators, including weight-for-length/height z-scores in young children, BMI-for-age z-scores in older children, and contextual clinical assessment (2–4).

Serum albumin has long been used in nutritional assessment, yet contemporary evidence shows that it is strongly influenced by inflammation, disease severity, and capillary permeability. Albumin may still carry clinically useful information, but it should not be viewed as a direct surrogate for nutritional stores (5).

Copeptin, the C-terminal fragment of the provasopressin peptide, is secreted in equimolar amounts with arginine vasopressin and is easier to measure because of its greater analytical stability. It reflects osmotic and hemodynamic stimuli as well as stress-related neuroendocrine activation (6, 7). Pediatric interest in copeptin has increased across a wide range of acute and chronic conditions (8), whereas direct data in undernutrition are limited; anorexia nervosa is among the few human conditions resembling severe undernutrition in which copeptin has been examined (9).

Despite growing interest in copeptin as a biomarker in pediatric disease, its potential role in children with undernutrition remains insufficiently characterized. The present study aimed to evaluate serum copeptin concentrations in children with undernutrition and to compare their discriminative performance with those of serum albumin. We also explored whether the combined use of copeptin and albumin could improve discrimination between undernourished children and healthy controls.

## Methods

### Study design and participants

This single-center cross-sectional case-control study was conducted between August 1, 2023 and January 1, 2024, in the outpatient pediatric clinic of Istanbul Haseki Training and Research Hospital, University of Health Sciences, Türkiye. The study was approved before initiation by the Clinical Research Ethics Committee of Istanbul Haseki Training and Research Hospital, University of Health Sciences (Decision No. 124-2023; July 26, 2023). Written informed consent was obtained from the parents or legal guardians of all participants. Children aged 1 month to 18 years were eligible for inclusion. Participants were classified into an undernutrition group and a healthy control group according to the criteria described below. All participants were Turkish nationals; ethnicity was not separately recorded. Detailed socioeconomic characteristics were not systematically recorded. The study was reported in accordance with the Strengthening the Reporting of Observational Studies in Epidemiology (STROBE) recommendations (10).

Participant recruitment was performed contemporaneously during the study period, with priority given to enrollment of eligible children with undernutrition. Eligible healthy controls of the same sex and a similar age were recruited from the same outpatient clinic, typically on the same or following day after enrollment of a child with undernutrition. Because eligible children with undernutrition were less frequent, a somewhat larger control group was intentionally recruited. Thus, controls were drawn from the same clinical setting and study period as the undernutrition group, but recruitment was not based on consecutive sampling of the entire outpatient population.

Children in the undernutrition group were identified at enrollment on the basis of age-appropriate anthropometric assessment used in routine clinical practice. For children younger than 5 years, nutritional status was assessed using weight-for-length/height z-scores, whereas for children aged 5 years and older, BMI-for-age z-scores were used, in accordance with WHO-aligned age-appropriate anthropometric criteria (3, 4). Undernutrition severity was categorized according to the degree of anthropometric deficit using the same age-appropriate z-score measure: mild undernutrition was defined as −2 < z-score < −1, moderate undernutrition as −3 < z-score ≤ −2, and severe undernutrition as z-score ≤ −3. These anthropometric criteria were used as operational definitions for study-group classification and were not intended to represent a comprehensive biological or functional definition of undernutrition.

Healthy controls were recruited from children presenting to the same outpatient clinic for routine evaluation and found to have no evidence of acute or chronic illness after clinical assessment and screening investigations.

Children with known chronic disease, suspected sepsis or acute infection, including C-reactive protein positivity, diabetes insipidus, Addison disease, electrolyte disturbance, elevated serum osmolality, urine specific gravity greater than 1.030, or evidence of chronic malnutrition/stunting were excluded. During screening, 30 children were excluded before final enrollment: 17 because of CRP positivity, 2 because of neurologic disease, 1 because of celiac disease, 2 because of elevated serum osmolality, and 8 because of chronic malnutrition/stunting (Figure 1).

**Figure 1:**
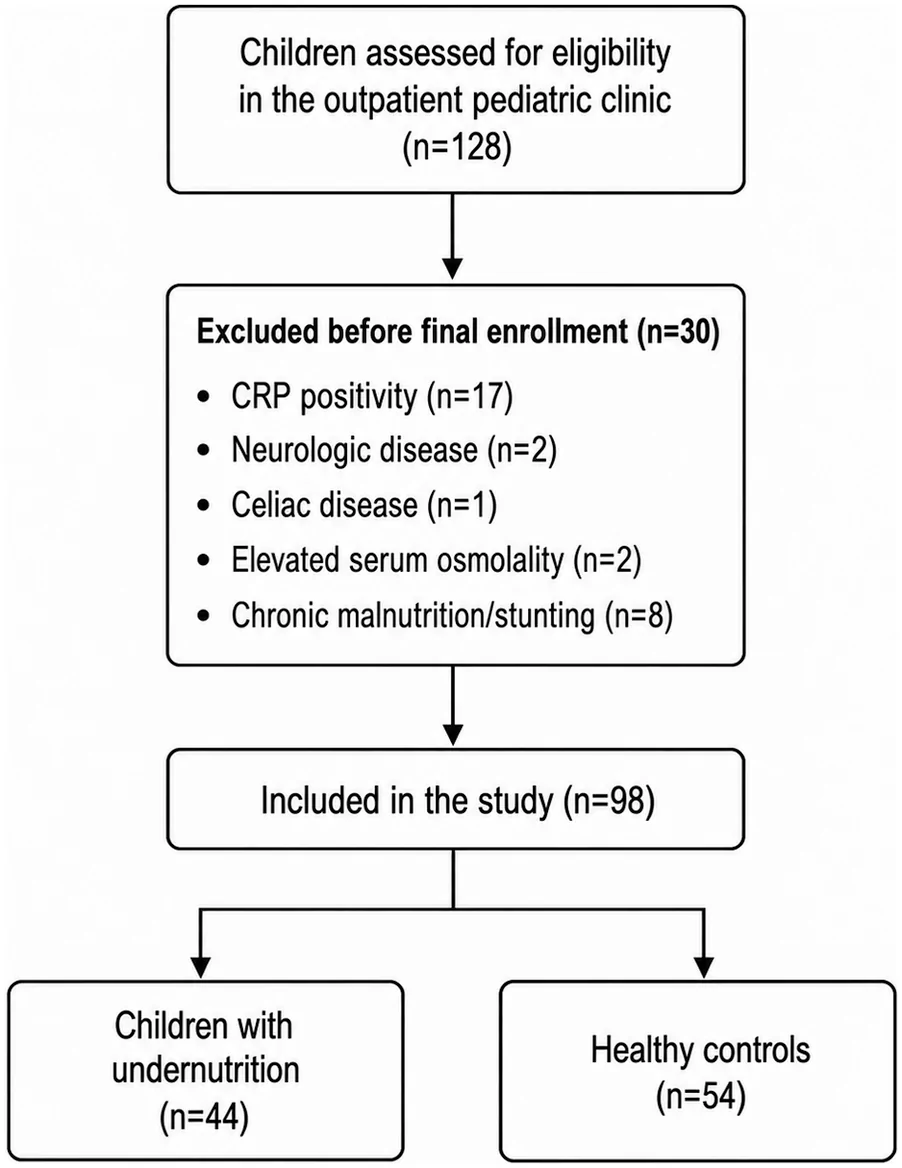
Flow chart of participant selection. A total of 128 children were assessed for eligibility. Thirty were excluded before final enrollment because of C-reactive protein positivity (*n* = 17), neurologic disease (*n* = 2), celiac disease (*n* = 1), elevated serum osmolality (*n* = 2), or chronic malnutrition/stunting (*n* = 8). The final study population comprised 98 children: 44 with undernutrition and 54 healthy controls. CRP, C-reactive protein.

### Anthropometric assessment

Anthropometric assessment included length/height, body weight, mid-upper arm circumference, and body mass index. All anthropometric measurements were performed by the same study physician (A.G.), who was trained in standardized pediatric anthropometric measurement techniques. Measurements were obtained without shoes and with the minimum amount of clothing compatible with privacy. In children younger than 2 years, recumbent length was measured using a seca 207 baby measuring rod (seca GmbH & Co. KG, Hamburg, Germany), and body weight was measured using a seca 354 digital baby scale (seca GmbH & Co. KG, Hamburg, Germany). In children aged 2 years and older, standing height was measured using an ADE MZ10023-1 stadiometer (ADE Germany GmbH & Co. KG, Hamburg, Germany), and body weight was measured using an ADE M320600-01 digital scale (ADE Germany GmbH & Co. KG, Hamburg, Germany). Mid-upper arm circumference was measured using a seca 201 ergonomic circumference measuring tape (seca GmbH & Co. KG, Hamburg, Germany) according to standard pediatric technique. The weighing devices underwent routine institutional calibration at predefined intervals. BMI was calculated as weight in kilograms divided by height in meters squared. Formal intraobserver reliability testing was not performed, and interobserver reliability could not be assessed because all measurements were obtained by a single observer.

The primary analysis was based on the prespecified case-control grouping used at enrollment. In addition, an exploratory secondary analysis was performed in which children with undernutrition were stratified into mild, moderate, and severe categories according to the degree of anthropometric deficit using the same age-appropriate WHO-aligned criteria (3, 4).

### Laboratory measurements

Venous blood samples were obtained at 09:00 after overnight fasting and approximately 8 h without water intake. Samples were centrifuged at 3,000 rpm for 15 min, aliquoted, and stored at −80 °C until analysis. Serum copeptin concentrations were measured in batch using a commercially available enzyme-linked immunosorbent assay kit (BT LAB/Bioassay Technology Laboratory, Shanghai, China) according to the manufacturer's instructions. The assay range was 1.12–44.94 pmol/L. Routine biochemical and hematological analyses, including serum albumin, electrolytes, renal function tests, CRP, and thyroid function tests, were performed in all participants. Urinalysis was also performed in all participants, and urine specific gravity greater than 1.030 was prespecified as an exclusion criterion.

For copeptin analysis, serum samples from participants in both study groups were assigned coded identifiers and transferred to the laboratory without information regarding nutritional group status. Samples from children with undernutrition and healthy controls were mixed within the analytical batches. Laboratory personnel performing the copeptin assays were therefore blinded to the anthropometric classification of the participants. Anthropometric classification had been established before copeptin assay results became available.

### Statistical analysis

Statistical analyses were performed using IBM SPSS Statistics for Windows, version 25.0 (IBM Corp., Armonk, NY, USA). Categorical variables were expressed as number (percentage), and continuous variables as mean ± standard deviation or median (interquartile range), as appropriate. Distributional assumptions were assessed using histograms, Q–Q plots, and the Shapiro–Wilk test, and homogeneity of variances was evaluated with Levene's test.

Between-group comparisons were performed using Student's t-test or the Mann–Whitney U-test for continuous variables, and the chi-square test or Fisher's exact test for categorical variables, as appropriate. Comparisons across more than two groups were performed using one-way ANOVA or the Kruskal–Wallis test, followed by Tukey's honestly significant difference test or Bonferroni-corrected pairwise comparisons, respectively, when the overall test was significant.

Receiver operating characteristic analyses, including comparison of the areas under the curve, were performed using jamovi (version 2.6.23). Multivariable logistic regression analyses were used to examine the independent associations of copeptin and albumin with undernutrition after adjustment for age and sex. Combined-model ROC analyses were based on predicted probabilities derived from multivariable logistic regression models. To assess the robustness of the biomarker associations to potential hydration- and osmoregulation-related biochemical confounding, an additional exploratory multivariable logistic regression model was fitted in the full cohort including copeptin, albumin, age, sex, serum sodium, urea, and creatinine. Because different age-appropriate anthropometric indicators were used in children younger than 5 years and those aged 5 years or older, exploratory age-stratified analyses were performed using the same age categories. Multivariable models additionally included age as a covariate to reduce potential confounding related to developmental differences across the broad pediatric age range. An additional sensitivity analysis compared children with moderate or severe undernutrition with healthy controls; ROC analyses and age- and sex-adjusted logistic regression were repeated in this restricted sample. A two-sided *p*-value <0.05 was considered statistically significant.

An *a priori* sample size calculation was performed using G*Power 3.1 (Heinrich Heine University Düsseldorf, Germany). Because no previous pediatric study had directly compared serum copeptin concentrations between children with undernutrition and healthy controls, the study by Tuli et al. was used as the closest available pediatric reference, and a large standardized between-group effect (Cohen's *d* = 0.80) was assumed for study planning (11). The original directional calculation indicated a minimum sample size of 70 participants (35 per group). Because the present analyses were conducted using two-sided tests, we additionally verified the corresponding two-sided requirement under the same effect-size, alpha (0.05), and power (95%) assumptions; this yielded 84 participants (42 per group).

## Results

### Participant characteristics

A total of 98 children aged 1 month to 18 years were included in the study: 44 children with undernutrition and 54 healthy controls. The mean age was 91.3 ± 69.4 months in the undernutrition group and 111.4 ± 55.5 months in the control group, with no significant difference when age was analyzed as a continuous variable (*p* = 0.113). The corresponding median ages were 91.5 months [IQR, 18.8–146.8] and 113.0 months [IQR, 74.0–148.0], respectively. Overall, 27 participants (27.6%) were younger than 5 years and 71 (72.4%) were aged 5 years or older. Children younger than 5 years were more frequent in the undernutrition group than in the control group (43.2% vs 14.8%, *p* = 0.003). Sex distribution was similar between the groups. Weight, weight SDS, height, height SDS, mid-upper arm circumference, BMI, BMI z-score, and weight-for-height percentage were all significantly lower in children with undernutrition than in healthy controls (Table 1).

**Table 1:** Demographic and anthropometric characteristics of children with undernutrition and healthy controls.

Variable	Healthy controls (*n* = 54)	Undernutrition (*n* = 44)	*p*
Girls, *n* (%)	25 (46.3)	27 (61.4)	0.137
Boys, *n* (%)	29 (53.7)	17 (38.6)	
Age <5 years, *n* (%)	8 (14.8)	19 (43.2)	0.003
Age ≥5 years, *n* (%)	46 (85.2)	25 (56.8)	
Age, months	111.4 ± 55.5	91.3 ± 69.4	0.113
Weight SDS	0.3 [−0.3; 1.0]	−1.9 [−2.4; −1.5]	<0.001
Height SDS	0.19 ± 1.02	−0.79 ± 1.08	<0.001
MUAC, cm	22.1 ± 5.6	16.7 ± 3.2	<0.001
BMI z-score	0.4 [−0.1; 0.9]	−1.9 [−2.5; −1.4]	<0.001
Weight-for-height, %	104.3 [101.2; 109.0]	83.7 [78.9; 88.2]	<0.001

Values are presented as mean ± SD, median [25th;75th percentile], or *n* (%), as appropriate. MUAC, mid-upper arm circumference; BMI, body mass index.

### Laboratory comparisons between groups

Among laboratory variables, red blood cell count, creatinine, and albumin were significantly lower in the undernutrition group, whereas lymphocyte count, potassium, and copeptin were significantly higher (Table 2).

**Table 2:** Laboratory findings in children with undernutrition and healthy controls.

Variable	Healthy controls (*n* = 54)	Undernutrition (*n* = 44)	*p*
WBC, 10^3^/µL	8763.8 ± 3689.5	8906.1 ± 4236.5	0.860
RBC, 10⁶/µL	4.7 ± 0.5	4.5 ± 0.5	0.030
Hemoglobin, g/dL	12.3 ± 1.4	11.9 ± 1.5	0.129
Hematocrit, %	37.2 ± 3.0	36.0 ± 4.1	0.089
Platelets,/µL	318000.0 [261000.0; 365000.0]	319000.0 [273250.0; 390750.0]	0.357
MCV, fL	78.2 ± 10.0	79.2 ± 9.8	0.612
Neutrophils,/µL	3590.0 [2890.0; 5390.0]	3120.0 [2355.0; 4467.5]	0.075
Lymphocytes,/µL	2921.9 ± 1319.8	3767.4 ± 1774.9	0.008
Glucose, mg/dL	90.5 [85.0; 98.5]	90.0 [83.5; 97.0]	0.982
Urea, mg/dL	21.2 ± 6.0	22.5 ± 9.9	0.453
Creatinine, mg/dL	0.5 ± 0.1	0.4 ± 0.2	0.034
ALT, U/L	14.5 [11.0; 17.8]	12.5 [10.0; 18.0]	0.630
AST, U/L	25.0 [19.2; 30.0]	24.0 [20.0; 30.8]	0.577
Sodium, mmol/L	138.3 ± 2.0	138.5 ± 2.0	0.559
Potassium, mmol/L	4.4 [4.2; 4.7]	4.6 [4.4; 4.9]	0.049
Chloride, mmol/L	101.5 ± 2.8	102.4 ± 2.1	0.075
Albumin, g/L	45.6 ± 2.8	41.1 ± 5.7	<0.001
Iron, µg/dL	71.0 [41.0; 83.0]	50.0 [39.0; 72.0]	0.242
TIBC, µg/dL	351.1 ± 45.5	367.4 ± 53.3	0.195
Ferritin, ng/mL	39.8 ± 37.3	38.9 ± 24.4	0.902
TSH, mU/L	2.2 [1.6; 3.2]	2.4 [1.7; 3.1]	0.851
Free T4, ng/L	12.2 ± 1.6	12.5 ± 1.4	0.351
Copeptin, pmol/L	2.5 ± 0.7	3.4 ± 1.3	<0.001

Values are presented as mean ± SD or median [25th;75th percentile], as appropriate. WBC, white blood cell count; RBC, red blood cell count; MCV, mean corpuscular volume; TIBC, total iron-binding capacity; TSH, thyroid-stimulating hormone.

### Exploratory discriminatory performance of copeptin and albumin

In ROC analysis, copeptin yielded an AUC of 0.729 (95% CI 0.622–0.835), whereas albumin yielded an AUC of 0.726 (95% CI 0.618–0.833), with no significant difference between the two ROC curves (DeLong *p* = 0.977) (Table 3; Figure 2). For descriptive exploratory purposes, the Youden-derived cut-off was ≥3.74 pmol/L for copeptin and ≤41.0 g/L for albumin. At these thresholds, copeptin showed 47.7% sensitivity and 94.4% specificity, whereas albumin showed 52.3% sensitivity and 94.4% specificity (Table 3).

**Table 3:** ROC-based discrimination and multivariable associations of serum copeptin and albumin with undernutrition.

A. ROC-based discrimination				
Biomarker	AUC (95% CI)	Youden-derived cut-off	Sensitivity (%)	Specificity (%)
Copeptin	0.729 (0.622–0.835)	≥ 3.74 pmol/L	47.7	94.4
Albumin	0.726 (0.618–0.833)	≤ 41.0 g/L	52.3	94.4
DeLong comparison of copeptin versus albumin ROC curves: *p* = 0.977.				

B. Multivariable logistic regression models			
Model term	OR	95% CI	*p*
Copeptin adjusted for age and sex	2.60	1.56 to 4.34	<0.001
Albumin adjusted for age and sex	0.79	0.69 to 0.89	<0.001
Copeptin in combined model	2.82	1.59 to 5.00	<0.001
Albumin in combined model	0.76	0.66 to 0.88	<0.001
Copeptin in hydration-adjusted model	2.74	1.52 to 4.93	<0.001
Albumin in hydration-adjusted model	0.76	0.65 to 0.88	<0.001

C. Model discrimination		
Model	AUC	Comparison
Age+sex+copeptin	0.763	Reference
Age+sex+albumin	0.750	Reference
Age+sex+copeptin+albumin	0.844	vs. age+sex+copeptin model, *p* = 0.033
		vs. age+sex+albumin model, *p* = 0.027

Combined-model ROC analyses were based on predicted probabilities derived from multivariable logistic regression models. The hydration-adjusted model included age, sex, copeptin, albumin, serum sodium, urea, and creatinine. AUC, area under the curve; CI, confidence interval; OR, odds ratio.

**Figure 2:**
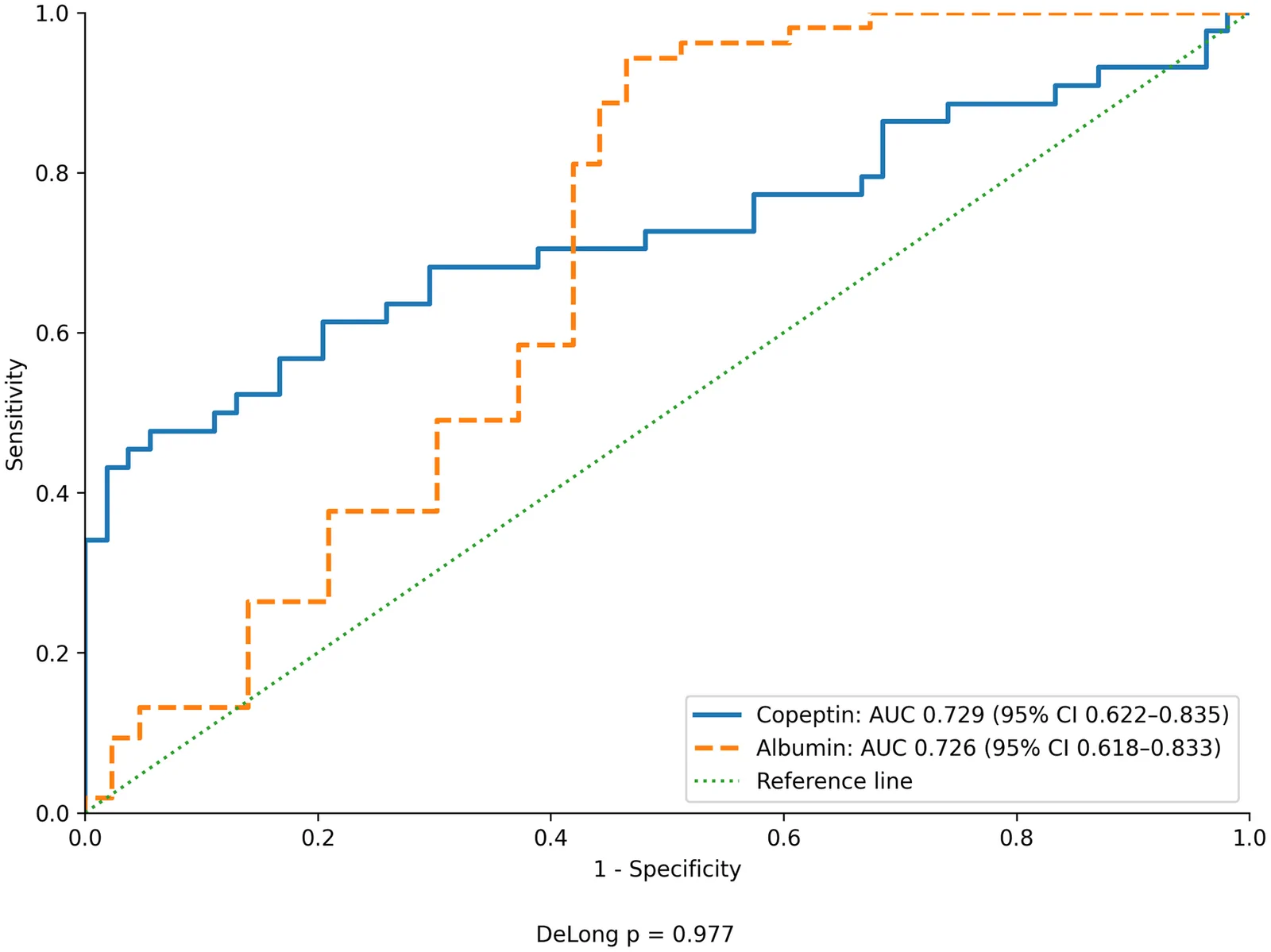
Comparative receiver operating characteristic curves for serum copeptin and albumin in discriminating children with undernutrition from healthy controls. The area under the curve was 0.729 (95% CI, 0.622–0.835) for copeptin and 0.726 (95% CI, 0.618–0.833) for albumin; the difference between the curves was not statistically significant (DeLong *p* = 0.977). ROC, receiver operating characteristic; AUC, area under the curve; CI, confidence interval.

### Multivariable and combined-model analyses

In multivariable logistic regression adjusted for age and sex, both copeptin and albumin remained independently associated with undernutrition. Copeptin was positively associated with case status, whereas albumin was inversely associated with case status. When both biomarkers were entered into the same model, both associations remained significant. In addition, the combined age-sex-copeptin-albumin model showed higher discrimination within the study sample than models including age and sex together with either copeptin or albumin alone (Table 3). In the full cohort, an additional exploratory model including copeptin, albumin, age, sex, serum sodium, urea, and creatinine yielded similar findings. Copeptin remained positively associated with undernutrition (OR 2.74; 95% CI 1.52–4.93; *p* < 0.001), whereas albumin remained inversely associated with undernutrition (OR 0.76; 95% CI 0.65–0.88; *p* < 0.001).

### Age-stratified and sensitivity analyses

In age-stratified analyses, copeptin and albumin yielded AUC values of 0.766 and 0.770, respectively, among children younger than 5 years. In children aged 5 years and older, the corresponding AUC values were 0.711 for copeptin and 0.692 for albumin (Table 4). These age-stratified estimates were exploratory and were not formally compared between age strata.

**Table 4:** Age-stratified, sensitivity, and severity-based analyses of serum copeptin and albumin in children with undernutrition and healthy controls.

A. Age-stratified ROC analyses					
Analysis subgroup		Copeptin AUC		Albumin AUC	
Children <5 years		0.766		0.770	
Children ≥5 years		0.711		0.692	

B. Sensitivity analysis comparing moderate/severe undernutrition with healthy controls					
Variable			Estimate	95% CI	*p*
Copeptin (age- and sex-adjusted OR)			2.74	1.26 to 5.96	0.011
Albumin (age- and sex-adjusted OR)			0.69	0.54 to 0.88	0.003
Copeptin AUC			0.695	0.522 to 0.868	0.030
Albumin AUC			0.824	0.677 to 0.972	<0.001

C. Exploratory severity-based analysis					
Variable	Healthy controls (*n* = 54)	Mild undernutrition (*n* = 31)	Moderate undernutrition (*n* = 9)	Severe undernutrition (*n* = 4)	*p*
Albumin, g/L	45.60 ± 2.7	41.83 ± 5.58	38.22 ± 5.43	41.75 ± 6.13	<0.001
Copeptin, pmol/L	2.50 ± 0.67	3.42 ± 1.23	3.07 ± 1.07	4.40 ± 2.06	<0.001

Values are presented as mean ± SD unless otherwise indicated. Age-stratified and moderate/severe-restricted analyses were exploratory. Severity categories were defined using age-appropriate WHO-aligned anthropometric criteria.

A sensitivity analysis comparing children with moderate or severe undernutrition with healthy controls yielded broadly similar results. In this subgroup, copeptin yielded an AUC of 0.695 (95% CI 0.522–0.868; *p* = 0.030), whereas albumin yielded an AUC of 0.824 (95% CI 0.677–0.972; *p* < 0.001). In age- and sex-adjusted models restricted to this subgroup, both copeptin and albumin remained significantly associated with undernutrition, supporting the robustness of the main findings despite the smaller sample size (Table 4).

### Exploratory severity-based analysis

In the exploratory severity-based analysis, 31 children were classified as having mild undernutrition, 9 as having moderate undernutrition, and 4 as having severe undernutrition. Relative to healthy controls, the severity-based groups showed significant overall differences in both serum albumin and copeptin concentrations (Table 4). Pairwise comparisons demonstrated that albumin concentrations were significantly lower in children with mild and moderate undernutrition than in healthy controls, whereas copeptin concentrations were significantly higher in children with mild and severe undernutrition than in controls. However, no significant differences in serum albumin or copeptin concentrations were observed among the mild, moderate, and severe undernutrition subgroups (Table 4).

## Discussion

### Principal findings and comparison with albumin

The principal finding of this study is that serum copeptin concentrations were higher in children with undernutrition than in healthy controls. Although copeptin did not outperform albumin as a standalone biomarker, both markers remained independently associated with case status in multivariable analysis, and their combination showed higher in-sample discrimination.

Serum albumin, a well-known visceral protein, has traditionally been considered a useful biochemical marker in nutritional assessment (12, 13). However, this traditional view has been increasingly challenged. Recent evidence suggests that albumin may be better interpreted as a nutrition-related inflammatory marker rather than a direct component of contemporary malnutrition definitions, and that reductions in serum albumin concentration may occur secondary to inflammation independently of underlying nutritional status (5). In keeping with previous reports, serum albumin levels were lower in children with undernutrition than in healthy controls in our study, although mean values remained largely within the conventional reference range.

### Biological interpretation of copeptin

The biological interpretation of copeptin in pediatric undernutrition is likely to be multifactorial. Copeptin reflects vasopressin release and is responsive to osmotic, hemodynamic, and stress-related stimuli (6, 7). Undernutrition has been linked to oxidative stress, immune dysfunction, and inflammatory activation (14, 15), all of which may contribute to copeptin elevation through neuroendocrine stress pathways. Experimental and translational evidence also supports interactions between inflammatory signaling, corticotropin-releasing hormone pathways, and activation of the vasopressin system (16). This interpretation is further supported by experimental studies linking vasopressin/copeptin activation to oxidative stress-related injury and by human studies showing elevated copeptin levels in disorders accompanied by increased oxidative stress, although a direct causal relationship has not been fully established (17, 18). In this context, the higher copeptin concentrations observed in children with undernutrition may reflect a nonspecific neuroendocrine response to metabolic, oxidative, or systemic stress associated with the undernourished state rather than a direct or specific signal of nutritional deficit itself.

To reduce potential between-group differences in thirst and hydration status, blood samples were collected from both undernourished children and healthy controls in the morning after overnight fasting and approximately 8 h without water intake. Overt hyperosmolality, electrolyte imbalance, and urine specific gravity greater than 1.030 were also prespecified exclusion criteria. Moreover, in an additional multivariable model including age, sex, copeptin, albumin, serum sodium, urea, and creatinine, both copeptin and albumin remained independently associated with undernutrition. These standardized sampling conditions, prespecified exclusions, and adjusted analyses reduce, but do not eliminate, the possibility that the observed copeptin difference was driven primarily by hydration- or osmoregulation-related factors. Nevertheless, copeptin should not be regarded as a nutrition-specific biomarker, and the present findings should be interpreted as exploratory.

### Exploratory discrimination and potential clinical implications

In exploratory ROC analysis, copeptin and albumin showed comparable discrimination between children with undernutrition and healthy controls, with no significant difference between their AUC values. To our knowledge, comparable pediatric data examining serum copeptin in undernutrition are lacking. Importantly, because the present study used a case-control design with selected healthy controls rather than a consecutive clinical population with diagnostic uncertainty, these ROC findings should not be interpreted as estimates of real-world diagnostic accuracy or clinical utility. Instead, they provide preliminary evidence that copeptin differs between the predefined anthropometric groups and may warrant prospective evaluation in clinically representative cohorts.

From a clinical perspective, copeptin should not be considered a replacement for anthropometric assessment or a standalone diagnostic test for pediatric undernutrition. Its potential value, if confirmed in prospective studies, may instead lie in providing complementary information on the neuroendocrine and physiological stress response associated with undernutrition. The higher discrimination observed when copeptin and albumin were considered together suggests that the two biomarkers may capture partly different biological dimensions. However, because the present case-control design does not provide validated estimates of real-world diagnostic accuracy, no copeptin threshold or clinical decision rule can be recommended on the basis of these data. Prospective studies in consecutively recruited and clinically representative pediatric populations are needed to determine whether copeptin adds clinically meaningful information beyond anthropometric assessment and conventional laboratory markers.

Practical considerations also differ substantially between the two biomarkers. Serum albumin is an inexpensive, widely available routine biochemical test, whereas copeptin measurement currently requires a dedicated immunoassay and is less readily available in routine pediatric practice. A formal cost-effectiveness analysis was beyond the scope of the present study; therefore, the potential incremental biological information provided by copeptin should be weighed against its greater analytical complexity and likely higher cost in future clinical studies.

### Age- and severity-related findings

The age-stratified analyses are also relevant given the broad developmental range of the study population and the use of different age-appropriate anthropometric indicators. Although both biomarkers showed somewhat higher AUC values in children younger than 5 years, these subgroup analyses were exploratory and the study was not powered for formal comparisons between age strata. The use of weight-for-length/height in younger children and BMI-for-age in older children may itself introduce heterogeneity despite both being age-appropriate anthropometric approaches. Accordingly, the subgroup findings should not be interpreted as evidence of an age-dependent biomarker effect. Future studies in more narrowly defined pediatric age groups are needed to determine whether the association between copeptin and undernutrition differs across developmental stages.

A sensitivity analysis comparing children with moderate or severe undernutrition with healthy controls yielded broadly similar results. In this subgroup, copeptin yielded an AUC of 0.695 (95% CI 0.522–0.868; *p* = 0.030), whereas albumin yielded an AUC of 0.824 (95% CI 0.677–0.972; *p* < 0.001). In age- and sex-adjusted models restricted to this subgroup, both copeptin and albumin remained significantly associated with undernutrition, supporting the robustness of the main findings despite the smaller sample size.

Additionally, we did not observe a significant difference in serum copeptin levels across undernutrition severity categories. This result should be interpreted cautiously because the number of children in the moderate and severe subgroups was small, which may have limited statistical power. Larger studies are needed to determine whether copeptin is associated with the severity of pediatric undernutrition.

### Strengths and limitations

This study has several strengths. It included a healthy control group, applied broad laboratory screening to reduce obvious confounding, and evaluated copeptin not only through unadjusted group comparisons but also through ROC and multivariable analyses. In addition, exploratory age-stratified and sensitivity analyses were performed to assess the consistency of the findings across different analytical frameworks.

Several limitations should be acknowledged. The study was conducted at a single center and had a cross-sectional design, which limits causal inference. The overall sample size was modest, and the moderate and severe undernutrition subgroups were small. Some subgroup and sensitivity analyses were therefore exploratory. Detailed socioeconomic characteristics were not systematically collected, which limited assessment of potential socioeconomic differences between the study groups. Although all anthropometric measurements were obtained by a single trained physician using standardized procedures, formal intraobserver reliability was not assessed. Furthermore, both albumin and copeptin are influenced by biological processes beyond nutrition itself, including inflammation, hydration, and neuroendocrine stress, which limits their specificity as isolated nutritional biomarkers. The cross-sectional nature of the study also precludes determination of whether increased copeptin is a consequence of nutritional deficit itself or reflects a nonspecific physiological response accompanying the anthropometric phenotype. The broad age range and the use of different age-appropriate anthropometric indicators in younger and older children introduced additional clinical heterogeneity despite age-adjusted and age-stratified analyses.

The findings of this study should also be interpreted in light of the anthropometric definition of the study groups. Weight-for-length/height and BMI-for-age are practical and standardized measures for identifying anthropometric deficits, but they do not fully characterize body composition, lean mass, duration of nutritional deprivation, metabolic consequences, or functional impairment. A substantial proportion of the undernutrition group had mild anthropometric deficit, and the study population included relatively older children compared with the age range in which clinically overt acute undernutrition is most commonly encountered. In addition, body-composition techniques such as bioelectrical impedance analysis, phase-angle assessment, or muscle ultrasonography were not available. Consequently, constitutional thinness or low lean mass cannot be completely excluded in some participants. Our results should therefore be interpreted as a biomarker signal associated with undernutrition rather than as evidence that copeptin identifies a comprehensive biological construct of malnutrition.

An additional limitation is the case-control sampling strategy. Participants were selected on the basis of predefined anthropometric group status rather than consecutively enrolled from a clinical population in whom nutritional status was diagnostically uncertain. This design may increase apparent separation between groups and introduces the possibility of spectrum bias (19). Accordingly, the ROC-derived measures in this study should be regarded as exploratory measures of discrimination within the study sample and should not be interpreted as validated estimates of diagnostic accuracy or directly extrapolated to routine clinical practice. Prospective studies with consecutive recruitment across the full clinical spectrum of pediatric nutritional status are required before the clinical diagnostic value of copeptin can be established.

## Conclusions

In conclusion, serum copeptin concentrations were higher in children with undernutrition than in healthy controls. Although copeptin did not outperform albumin as a standalone biomarker, both markers were independently associated with case status, and their combined use showed higher discrimination within the study sample. Copeptin should not be interpreted as a nutrition-specific marker on the basis of these findings; rather, the observed elevation may reflect physiological stress associated with the anthropometric phenotype. These findings support further prospective investigation of copeptin as a potential adjunctive biomarker in clinically representative cohorts with more comprehensively characterized undernutrition.

## Data Availability

The datasets generated and analyzed during the current study are not publicly available because they contain potentially identifiable participant-level information, but they are available from the corresponding author on reasonable request and subject to institutional approval.
